# Molecular Alterations Caused by Alcohol Consumption in the UK Biobank: A Mendelian Randomisation Study

**DOI:** 10.3390/nu14142943

**Published:** 2022-07-19

**Authors:** Felix O’Farrell, Xiyun Jiang, Shahad Aljifri, Raha Pazoki

**Affiliations:** 1 Division of Biomedical Sciences, Department of Life Sciences, College of Health and Life Sciences, Brunel University London, Uxbridge UB8 3PH, UK; felix.ofarrell@gmail.com (F.O.); xiyunjiang@yahoo.com (X.J.); shahad.aljifri@gmail.com (S.A.); 2 Department of Epidemiology and Biostatistics, School of Public Health, St Mary’s Campus, Norfolk Place, London W2 1PG, UK

**Keywords:** Mendelian randomisation, alcohol consumption, UK biobank, Phenome wide association studies, biomarker

## Abstract

Alcohol consumption is associated with the development of cardiovascular diseases, cancer, and liver disease. The biological mechanisms are still largely unclear. Here, we aimed to use an agnostic approach to identify phenotypes mediating the effect of alcohol on various diseases. Methods: We performed an agnostic association analysis between alcohol consumption (red and white wine, beer/cider, fortified wine, and spirits) with over 7800 phenotypes from the UK biobank comprising 223,728 participants. We performed Mendelian randomisation analysis to infer causality. We additionally performed a Phenome-wide association analysis and a mediation analysis between alcohol consumption as exposure, phenotypes in a causal relationship with alcohol consumption as mediators, and various diseases as the outcome. Results: Of 45 phenotypes in association with alcohol consumption, 20 were in a causal relationship with alcohol consumption. Gamma glutamyltransferase (GGT; *β* = 9.44; 95% CI = 5.94, 12.93; *P_fdr_* = 9.04 × 10^−7^), mean sphered cell volume (*β* = 0.189; 95% CI = 0.11, 0.27; *P_fdr_* = 1.00 × 10^−4^), mean corpuscular volume (*β* = 0.271; 95% CI = 0.19, 0.35; *P_fdr_* = 7.09 × 10^−10^) and mean corpuscular haemoglobin (*β* = 0.278; 95% CI = 0.19, 0.36; *P_fdr_* = 1.60 × 10^−6^) demonstrated the strongest causal relationships. We also identified GGT and physical inactivity as mediators in the pathway between alcohol consumption, liver cirrhosis and alcohol dependence. Conclusion: Our study provides evidence of causality between alcohol consumption and 20 phenotypes and a mediation effect for physical activity on health consequences of alcohol consumption.

## 1. Introduction

Alcohol use is responsible for 5.1% of the global burden of disease [1] and is considered the main contributor to alcohol liver disease (ALD). Despite a general understanding of the link between alcohol consumption and diseases, the causal associations and mediatory mechanisms are less clear and health effects of moderate drinking remain a hazy area of research [2]. A current advice of drinking no more than 14 units of alcohol a week [3] is said to only minimize morbidity and mortality risks and does not imply any “safe” drinking level [4]. Previous positions regarding positive alcohol or wine intake are now being revised [4]. Currently, guidelines related to the risk of alcohol consumption are inconsistent and cause uncertainty about dangers and consequences of alcohol consumption [1].

Recent studies highlight the need for research to identify and quantify phenotypes impacting the risk of alcohol consumption, including frequency of alcohol, dietary combinations [5], and, specifically, different alcoholic beverages (wine, beer, spirits, and others). Recently, Jani and colleagues investigated the large dataset of the UK Biobank and estimated a seven-year predicted probability of major adverse cardiovascular events according to alcoholic beverage type [6]. They found that the consumption of spirits had the highest risk of a cardiovascular events, followed by beer/cider in second position, and white/red wine demonstrating the lowest risk of cardiovascular events [6]. The study highlighted the importance of alcohol consumption on the risk of diseases. It underscored the importance of research to identify molecular changes that occur with drinking various alcoholic beverages, to provide insight into pinpointing pathways involved in increasing such risk.

Identification of molecular changes and causal biomarkers in the pathway between alcohol consumption and alcohol related diseases could enlighten the mechanisms involved in risks of alcohol consumption. It could also facilitate utility of these biomarkers to better identify individuals at high risk of developing alcohol-related diseases. These high-risk individuals could then be targeted to receive public health interventions. Our study aimed to identify molecular changes in a causal link with alcohol consumption and alcohol-related diseases. We used Phenome wide association (PheWAs) and Mendelian randomisation (MR) methods.

PheWAs examine correlation between an exposure (a variant or phenotype of interest) with an array of outcomes (the phenome) [7]. The power of PheWAS is determined by the sample size and variety of clinical information present in the database [8]. The current gold-standard to perform PheWAS are large and comprehensive electronic health record datasets [9].

The PheWAS method quantifies associations between exposures and outcomes and is unable to assess causal links. MR studies investigate causality between instruments and often follow PheWAS to further interrogate a suggested association. MR studies operate upon the fact that most genetic variants are inherited randomly from parents and can be used as a randomisation tool to mimic randomised clinical trials [10]. Owing to the random nature of genetic inheritance, MR studies are less biased due to issues such as reverse causality and confounding [11]. Genetic variants that are associated with phenotypes (found from association studies) can be used as exposure instruments to test for causal associations against a given outcome [10].

Here, we applied a multi-stage design to identify phenotypes in a causal pathway between alcohol consumption and alcohol-related diseases. Using a combination of agnostic approaches, PheWAS, and MR analyses, we investigated over 7800 phenotypes in the UK biobank cohort for association and causal links with alcohol consumption and alcohol related diseases.

## 2. Methods

We used data from the UK biobank (*N* = 223,728), which is a large biomedical database with genotypic and phenotypic data on a wide range of health-related outcomes for over 500,000 individuals. Participants between age 40 and 69 and living within 30 miles of one of the 22 UK biobank assessment centres were invited to take part. The UK biobank has full ethical approval by the UK NHS National Research Ethics Service [9]. All participants in this study gave consent for their data to be used [12,13].

**Figure 1:**
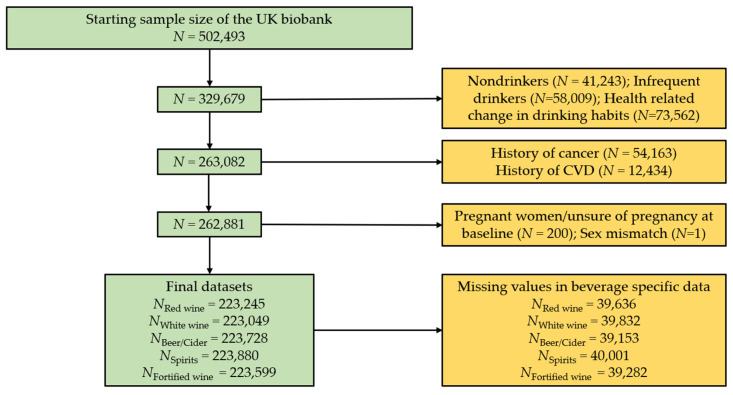
Overview of included and excluded participants. Green colour shows the remaining participants. Amber represents excluded participants. CVD, cardiovascular disease.

Participants who stated drinking of at least one of the alcoholic beverages red wine, white wine, beer/cider, spirits, and fortified wine were included in the current analysis (Figure 1). Participants who withdrew consent were removed from the dataset (*N* = 109), leaving a starting total of *N* = 502,493. Individuals who completed the UK biobank touchscreen questionnaire and declared they do not drink (*N* = 41,243), only drink on special occasions (*N* = 58,009), or changed drinking habits due to health reasons as described to participants in a touch screen questionnaire (e.g., illness or ill health, upon doctor’s advice, or out of health precaution; *N* = 73,562) were removed from this analysis. This ensured our analyses focused on individuals who normally consume alcohol. Using the same questionnaire, individuals who had serious co-morbidities, at baseline such as self-reported cancer (*N* = 54,163), and cardiovascular disease (*N* = 12,434) were also excluded. Participants who had missing data for the alcohol phenotypes of interest, pregnant women (*N* = 200), and individuals who had missing sex data (*N* = 1) were excluded. Individuals who passed the exclusion criteria but did not have beverage specific data were removed from this analysis. This slightly varied depending on the beverage type (Figure 1). Final beverage-specific datasets used in our analysis include red wine (*N* = 223,245), white wine or champagne (*N* = 223,049), beer or cider (*N* = 223,728), fortified wine (*N* = 223,599), and spirits (*N* = 222,880).

**Figure 2:**
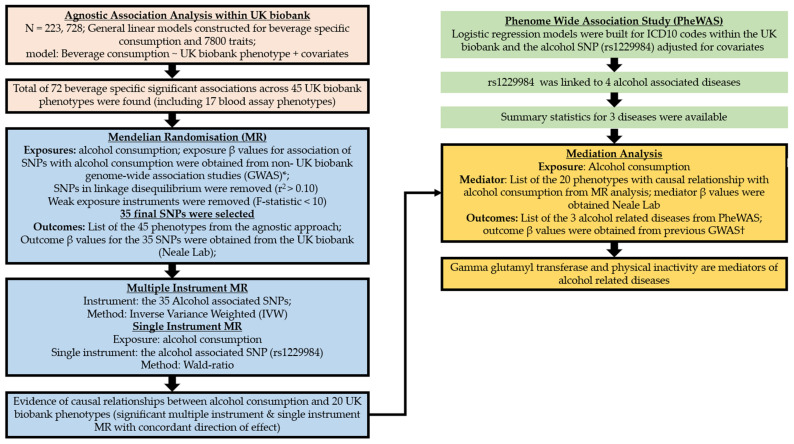
Overview of the study design. * Beta values for exposure were obtained from the non- UK biobank part of the summary statistics provided by Liu and colleagues (multiple instrument MR) [15] and Jorgenson and colleagues (single instrument MR) [16]. † In the mediation analysis, outcome beta values were obtained from Olfson and colleagues [17] and Butch and colleagues [18]. SNP, single nucleotide polymorphism.

The overview of the study design is presented in Figure 2. Data collection was performed centrally by the UK biobank. Between 2006 and 2010, touchscreen questionnaires and in-person interviews were conducted by UK biobank in one of their 22 UK-wide assessment centres. These baseline assessments include lifestyle choices, environmental factors, along with personal and family medical history. UK biobank participants also gave blood and urine samples [9]. All biological samples were obtained and analysed using the protocol outlined by the UK biobank [14]. Diagnosis of diseases were made based on the International Classification of Diseases (ICD 10) coding within the UK biobank data (https://biobank.ndph.ox.ac.uk/showcase/field.cgi?id=41203, accessed on 20 November 2021).

Self-reported information describing the weekly frequency of different kinds of alcoholic beverages have been collected in the UK biobank. Participants specified their consumption based on the number of glasses of red wine, white wine/champagne, and fortified wine in an average week. The number of pints of beer/cider consumed per week and measures of spirits or liquors consumed per week were collected to assess consumption of beer/cider and spirits.

### 2.1. Beverage Specific Agnostic Association Analyses

We performed an initial agnostic analysis in which over 7800 phenotypes and circulatory biomarkers from the UK biobank were investigated for association with drinking alcoholic beverages. The weekly consumption of red wine, white wine/champagne, beer/cider, spirits, and fortified wine were used as the outcome (dependent variables) in each association analysis; this means that we performed five association analyses regressing the consumption of each of the five alcoholic beverages on to every UK biobank phenotype (independent variable). This lead to construction of five general linear models for each of the 7803 UK biobank phenotypes. We adjusted the linear regression models for potential confounders (age, sex, Townsend deprivation index, genetic and ethnic background, smoking status, and diabetes).

To test the significance for each model we calculated an empirical *p*-value using a 10,000-iteration permutation test [19]. A permutation test calculates the probability (empirical *p*-value) that the observed *p*-value is driven by chance. To calculate the empirical *p*-value, we randomly sampled the outcome variable and performed an association analysis that calculates the *p*-values driven by chance (permuted *p*-value). This procedure was repeated 10,000 times, which generated 10,000 permuted *p*-values for each observed regression model. The number of times permuted *p*-values were less than the observed *p*-value was divided by the total number of iterations using the equation below:

$$P_{e}=\frac{n+1}{s+1}$$

where *P_e_* is the empirical *p*-value, *n* is the number of permuted *p*-values less than the observed *p*-value, and *s* is the number of iterations of the permutation test (*N* = 10,000). If the empirical *p*-value was less than 0.05, it indicated that less than 5% of the permuted *p*-values for the model were smaller than the observed *p*-value. This means that the observed *p*-value obtained from our linear regression model was not driven by chance and therefore the model was considered significant [19]. For significant models, we additionally estimated the percentage of variance in the alcoholic beverage consumption explained by each phenotype.

### 2.2. Two Sample MR

MR analysis uses genetic variants to explore causal relationships between an exposure and outcome. To infer causality between the effect of alcohol consumption and other phenotypes, we performed two sample MR analyses, where genetic associations are typically obtained from two independent studies (samples). In our MR analyses, alcohol consumption was used as the exposure, which means that genetic variants associated with alcohol consumption were used as an instrumental variable. All phenotypes that were significantly associated with alcoholic beverages consumption in our initial agnostic association analysis were tested for causality against alcohol consumption using MR analysis. The analysis was performed using two methods: (1) multiple instrument MR, where we combined multiple alcohol associated genetic variants [15] or (2) single instrument MR, where the single rs1229984 alcohol genetic variant was used for MR analysis [16].

#### 2.2.1. Instrument Selection (Multiple Instrument MR)

We obtained genetic association statistics (*β* values) from previously performed genome-wide association analyses (GWAS) performed to assess the effect of genetic variants in the form of single nucleotide polymorphism (SNP) on alcohol consumption [15,16]. Liu and colleagues performed meta-analyses across multiple alcohol consumption GWAS including the UK biobank. The *β* value for each of the genetic variants associated with alcohol consumption from the study of Liu and colleagues was used as an instrumental variable (alcohol consumption was defined as the exposure; see above). To avoid sample overlap with the UK biobank, we selected the *β* values for alcohol consumption from one of the meta-analyses by Liu and colleagues that excluded UK biobank data.

Weak instruments are genetic variants that do not capture enough variance in an exposure and introduce bias into a MR analysis [20]. To account for weak instrument bias, the *β* values were used to calculate the phenotype proportion of variance explained (R^2^) and the F-statistic for each instrument (Supplementary Table S1). The F-statistic is a measure of the association between the genetic variant and the exposure. Any instrument scoring an F-statistic of less than 10 was removed from the analysis [21].

In addition, genetic variants that were in linkage disequilibrium with other variants (indicated by r^2^ < 0.1) were identified using the European population data from the 1000 genomes project [22] and were excluded from the instrument list.

To obtain the *β* value for genetic variants associated with outcomes (shortlisted phenotypes from our earlier agnostic models), we used summary statistics from Neale Lab’s UK biobank GWAS studies (http://www.nealelab.is/ accessed on 20 January 2021). Neale lab has performed linear regression GWAS on almost all UK biobank phenotypes, making it a consistent set of summary statistics to use for our MR analyses.

The R package TwoSampleMR was used to harmonise the exposure and outcome effect estimates [23]. The Inverse Variance Weighting (IVW) method implemented in the MR-PRESSO package was used to perform the MR analyses and identify outlier genetic variants responsible for horizontal pleiotropy (a source of bias in MR analysis) and remove them from the analysis [24]. To account for multiple testing, a false discovery rate (FDR) of 0.05 was used to adjust the *p*-values from all MR analyses.

#### 2.2.2. Single-Instrument MR Analysis

As a sensitivity analysis for our causal inference, we performed a single-instrument MR analysis in which the *β* value for the association of rs1229984 with alcohol consumption was obtained from the study by Jorgenson and colleagues [16]. rs1229984 occurs in the *ADH1A* gene, as is known to be a functional non-synonymous (Arg48His) genetic variant for alcohol consumption [25]. The *ADH1B* gene encodes for an enzyme responsible for oxidising alcohol [26]. The Wald ratio method was used for our single-instrument MR analysis.

Results that consistently demonstrated causal relationship with the same direction of effect across both multiple and single-instrument MR analysis were considered significant.

### 2.3. Phenome-Wide Association Analysis

We performed PheWAs on rs1229984 to identify association of this genetic variant with alcohol-related diseases within the UK biobank. We used Logistic regression models within the R package PheWAS. Genotypes for the rs1229984 were extracted from individual level data of the UK biobank using plink [27]. A list of diagnosed diseases and conditions from hospital episode statistics were available within the UK biobank data in the form of ICD10 codes. We adjusted for the same potential confounders that were included in our agnostic analyses. To account for multiple testing, a Bonferroni correction was implemented.

### 2.4. Mediation Analysis

We performed a mediation analysis to investigate whether the UK Biobank phenotypes could mediate the effect of alcohol consumption on alcohol-related diseases. 

The mediation analysis included two steps. In the first step, we tested for the causal link between alcohol consumption (the exposure) and the UK Biobank phenotypes (the mediators); this was described above (see Section 2.1 and Section 2.2). In the second step, we tested for the causal link between the mediators and alcohol-related diseases (the outcome). The list of alcohol-related diseases that we found associated with rs1229984 in our PheWAS analysis (see Section 2.3) were used as outcome of the second step. To obtain outcome *β* values, we used previously published GWAS on alcohol-related diseases [17,18,28]. The mediator *β* values were obtained from Neale lab and used as the instruments in the second step. SNP selection methods that were described in the MR instrument selection section (see Section 2.2.1) were also applied to the mediation analysis. The Sobel test was used to calculate the indirect effects in the mediation analysis [29]. 

## 3. Results

**Figure 3:**
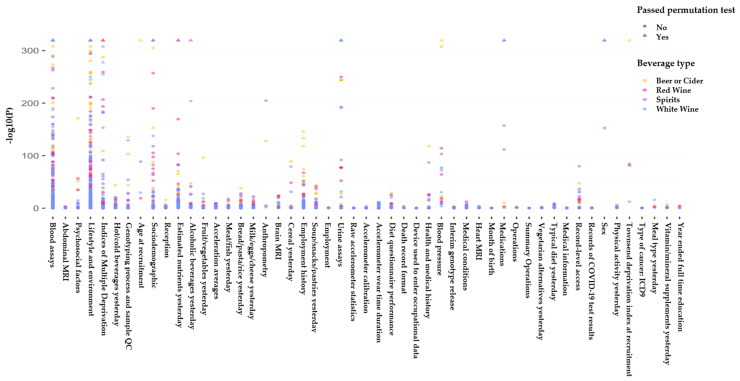
Overview of the agnostic association analyses. The Manhattan plot illustrates negative log10 observed *p*-values for general linear models created for the association of UK biobank phenotypes with consumption of various alcoholic beverages (e.g., beer, cider, spirit, red, white, and fortified wine). UK biobank phenotypes have been categorized into groups for the purpose of visualization in this figure. To account for multiple testing, significant models were selected using the permutation method, which estimates the likelihood of the observed associations to be driven by chance. We simulated chance finding by repeating each association test 10,000 times with the outcome that was randomly sampled. If more than 5% of the random associations led to *p*-values that were less that the observed *p*-value (obtained from our UK Biobank data), we concluded that the observed *p*-value is likely to be driven by chance and therefore we did not accept them as significant models. Models that passed the permutation test are plotted as triangles and models that did not pass the permutation test are plotted as circles. Beer/cider results are plotted in yellow, red wine results are plotted in pink, spirit results are plotted in purple, and white wine results are plotted in blue.

Our data for analysis included 223,728 individuals from the UK biobank of whom 48% were males (Table 1). The average age in the cohort used in our analysis was 55.5 (±8.01) and the average body mass index (BMI) was 26.9 (±4.33). We found 45 phenotypes (Figure 3) that were significantly associated with consumption of at least one alcoholic beverage (beer/cider, white wine, red wine, fortified wine, and spirits).

**Figure 4:**
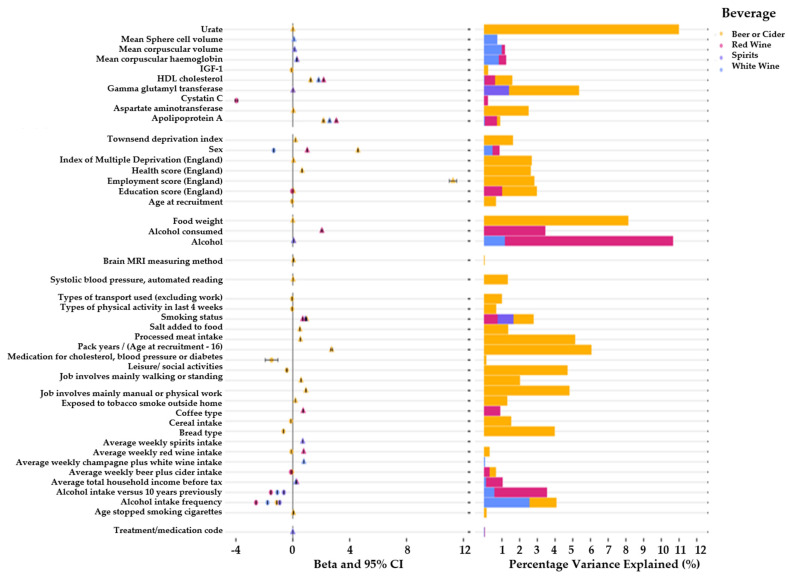
Overview of leading associations between consumption of alcoholic beverages and various phenotypes within the UK biobank. The left panel illustrates the effect estimates and confidence intervals for the leading associations. The right panel illustrates percentage variance explained for association of various alcoholic beverage consumption and UK biobank phenotypes. Beer/cider results are plotted in yellow, red wine results are plotted in pink, spirit results are plotted in purple, and white wine results are plotted in blue.

We observed an association between gamma glutamyl transferase (GGT) with increased consumption of beer/cider (*β* = 0.02; 95% CI = 0.019, 0.021; *p* < 1.0 × 10^−300^; r^2^ = 5.3%) and spirits (*β* = 0.01; 95% CI = 0.009, 0.011; *p* < 1.0 × 10^−300^; r^2^ = 1.39%). The Insulin-like Growth Factor (IGF-1) indicated a negative association with beer/cider consumption (*β* = −0.09; 95% CI = −0.094, −0.086; *p* < 1.0 × 10^−300^; r^2^ = 0.21%) (Figure 4). Participants of the UK biobank demonstrated a significant increase in the levels of apolipoprotein A1 (apo-A1) in their blood assays if they reported a higher weekly consumption of beer or cider (*β* = 2.2; 95% CI = 2.10, 2.30; *p* < 1.0 × 10^−300^; r^2^ = 0.89%), white wine (*β* = 0.09; 95% CI = 0.086, 0.094; *p* < 1.0 × 10^−300^; r^2^ = 2.89%), and red wine (*β* = 3.1; 95% CI = 3.002, 3.198; *p* < 1.0 × 10^−300^; r^2^ = 0.71%). We additionally observed an association between cystatin c levels in the blood and red wine consumption (*β* = −4.0; 95% CI = −4.20, −3.80; *p* < 1.0 × 10^−300^; r^2^ = 0.20%). Mean corpuscular haemoglobin (MCH) showed an association with white (*β* = 0.27; 95% CI = 0.27, 0.27; *p* < 1.0 × 10^−300^; r^2^ = 0.89%) and red wine (*β* = 0.30; 95% CI = 0.29, 0.31; *p* < 1.0 × 10^−300^; r^2^ = 1.23%). The mean corpuscular volume (MCV) was associated with white wine (*β* = 0.12; 95% CI = 0.116, 0.124; *p* < 1.0 × 10^−300^; r^2^ = 1.25%) and red wine (*β* = 0.12; 95% CI = 0.114, 0.126; *p* < 1.0 × 10^−300^; r^2^ = 1.16%). The mean sphered cell volume (MCSV) was associated with white wine (*β* = 0.09; 95% CI = 0.086, 0.094; *p* < 1.0 × 10^−300^; r^2^ = 1.04%).

We also observed evidence of an association between the systolic blood pressure with beer/cider consumption (*β* = 0.02; 95% CI = 0.019, 0.021; *p* < 1.0 × 10^−300^; r^2^ = 2.4%).

In our MR analysis using the IVW method, alcohol consumption demonstrated association with the liver enzyme GGT (*β* = 9.7; 95% CI = 5.8, 13.6; *p* ≤ 0.0001; Table 2). We also observed significant associations between alcohol consumption and multiple lifestyle choices. These included dietary factors such as an individuals’ preference to wholemeal or wholegrain (*β* = −0.05; 95% CI = −0.09, −0.02; *p* = 0.006) and white bread preference (*β* = 0.05; 95% CI = 0.02, 0.09; *p* = 0.002). We additionally observed significant associations between alcohol consumption and MSCV (*β* = 0.2; 95% CI = 0.15, 0.31; *p* ≤ 0.0001), MCV (*β* = 0.3; 95% CI = 0.18, 0.36; *p* ≤ 0.0001), and MCH (*β* = 0.3; 95% CI = 0.18, 0.36; *p* ≤ 0.0001).

The results from the PheWAS analysis (Table 3) demonstrated significant associations between alcohol SNP (rs1229984) and general alcohol-related diseases (*β* = 0.24; 95% CI = 0.16, 0.32; *p* = 4.78 × 10^−10^), alcohol dependency (*β* = 0.26; 95% CI = 0.16, 0.36; *p* = 2.52 × 10^−8^), alcoholic liver damage (*β* = 0.27; 95% CI = 0.15, 0.39; *p* = 3.47 × 10^−6^), and enthesopathy (*β* = −0.06; 95% CI = −0.08, −0.04; *p* = 1.05 × 10^−5^). 

**Figure 5:**
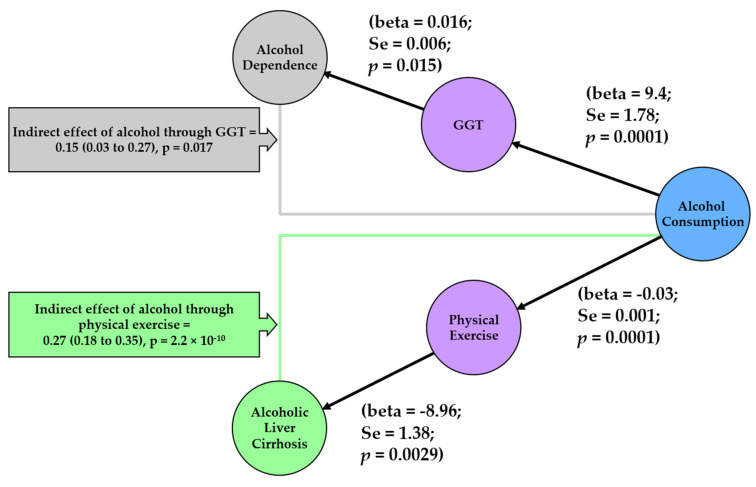
Network to summarise mediators of alcohol consumption and alcohol related diseases. Each node represents a phenotype that was included in mediation analysis. The color of the nodes indicates the source from which the test statistics for genetic associations were obtained. Blue refers to the GWAS by Liu and colleagues [15], purple refers to the UK biobank GWAS by Neale lab, green refers to the GWAS by Buch and colleagues [18], and grey refers to the GWAS by Olfson and colleagues [17]. Results from the MR analysis between each pair of phenotypes is depicted on the edges. For indirect effect, beta from the Sobel method is given together with the 95% CI in brackets and the *p* value for the Sobel method’s *z* value. beta, effect estimate from Mendelian Randomisation; Se, standard error; *p*, *p*-value; GGT, Gamma Glutamyl transferase.

In our mediation analysis (Figure 5), we observed that GGT mediated the effect of alcohol consumption on alcohol dependence (*β_Sobel_* = 0.15; 95% CI = 0.03, 0.27; *p* = 0.017). In addition, physical inactivity mediated the effect of alcohol consumption on alcoholic liver cirrhosis (*β_Sobel_* = 0.27; 95% CI = 0.18, 0.35; *p* = 2.21 × 10^−10^). 

## 4. Discussion

Here, we found evidence of 20 causal relationships between UK biobank phenotypes and alcohol consumption. We also identified: (1) GGT as a possible mediator of alcohol consumption’s effect on alcohol dependence, and (2) low levels of physical activity as a possible mediator of alcohol liver cirrhosis. The identification of these risk factors between alcohol consumption and various diseases may help with identification of individuals who are at a higher risk for developing alcohol-related diseases. 

We observed that the liver enzyme GGT demonstrated a significant association with alcohol consumption (i.e., higher consumption of spirits, beer, or cider is linked to a higher serum level of GGT). The association was further supported by the MR analyses. Furthermore, we identified GGT as a possible mediator of the effect of alcohol consumption on alcohol dependence. These findings are in line with previous epidemiological evidence and goes further to show the causality of this relationship. A four-year prospective study with 6846 male participants observed that alcohol consumption was associated with the raised blood levels of multiple liver enzymes, including GGT [30]. Furthermore, one study found that moderate alcohol drinking (which was defined as less than 40 g ethanol per day) raised GGT but did not significantly raise other liver enzymes, such as AST [31]. Our analysis was corrected for BMI as it was previously found that BMI has a larger effect on liver enzymes than alcohol consumption alone [30].

We also demonstrated that physical inactivity is a mediator between alcohol consumption and alcoholic liver cirrhosis. The results indicate that higher alcohol consumption is linked to lower physical activity, which in turn is linked to higher liver cirrhosis (Figure 5). This could possibly be due to the impact that physical inactivity has on obesity and thus liver fat (a contributor to non-alcoholic fatty liver disease and a source for liver cirrhosis). Whilst alcohol consumption on its own has a direct impact on liver cirrhosis [32], our study demonstrates that it may work through another pathway by lowering physical activity and indirectly impacting on liver cirrhosis.

Our initial agnostic analysis found associations between erythrocyte phenotypes (e.g., MSCV, MCV, and MCH) and alcohol consumption. Our MR analyses demonstrated a positive causal relationship between wine consumption and erythrocyte characteristics. The non-alcoholic properties of red wine have been found to act as an antioxidant [33]. Additionally, grape and wine products contain substantial amounts of iron [34], which is a fundamental trace element in the production of erythrocytes. Whilst severe alcoholism has been associated with anaemia and raised reticulocyte count and size [35,36], Toth and colleagues demonstrated increased hematologic parameters in 39 healthy non-smoking volunteers after exposure to red wine [37].

Our agnostic analysis highlighted that beer/cider consumption was linked to hyperuricemia. Excess alcohol consumption is well documented in epidemiological studies to be linked to hyperuricemia [38,39]. Specifically, beer and spirits are linked to hyperuricemia compared with other types of alcoholic drinks [40,41]. Our study is in line with these epidemiological studies.

Our analysis benefited from the large sample size and the rich phenotyping of the UK biobank cohort [9]. This improves our statistical power to detect phenotypes associated with alcoholic beverage consumption [42]. Our beverage-specific analyses, tied with the large sample size of the UK biobank, highlighted several phenotypes associated with the consumption of specific beverages. Another strength of our study lies in the number of MR studies we performed to aid us in identifying the large number of phenotypes in a causal relationship with alcohol consumption. Furthermore, our agnostic approach reduces bias and ensures identification of novel phenotypes associated with alcohol consumption [43]. Finally, mediation analysis gave us a better understanding of the relationships between alcohol associated phenotypes and the alcohol related diseases [44].

Our MR analysis was limited by the lack of beverage-specific genetic instruments (e.g., instruments specific to beer/cider or spirit consumption). This would have allowed us to test for causal links specific to these beverages and not be limited to instruments associated with general alcohol consumption. Another limitation of the study is that genetic components have been found to account for a small amount of variance in alcohol consumption [45]. In our study, we had 34 self-reported phenotypes. Due to recall bias, self-reported data (e.g., physical exercise) are less generalizable compared to a measured phenotype (e.g., BMI). The use of genetic test statistics for alcohol consumption from studies independent of the UK Biobank improves the robustness of our findings. Our conservative approach in performing multiple stages of analyses, starting from agnostic association analysis, MR analysis, PheWAS, and mediation analysis that made use of various data sources, ensures robustness of the results presented.

## 5. Conclusions

We used an agnostic approach to identifying the causal factors associated with the consumption of alcoholic beverages. We specifically highlighted that the consumption of beer and spirits might cause different molecular changes than the consumption of red and white wine. Our findings imply that liver function and physical inactivity may mediate the effect of alcohol consumption on alcohol dependence and alcohol cirrhosis. Further research in this area is essential as individuals who are physically less active and those with higher level of liver enzyme GGT might be more susceptible to adverse effects of alcohol consumption. 

## Figures and Tables

**Table 1:** Baseline characteristics of the population for analysis.

Characteristics	Red Wine Dataset (*N* = 223,245)	White Wine/Sparkling White Wine Dataset (*N* = 223,049)	Beer or Cider Dataset (*N* = 223,728)	Spirits Dataset (*N* = 222,880)	Fortified Wine Dataset (*N* = 223,599)
Age-yr	55.5 (±8.01)	55.5 (±8.01)	55.5 (±8.01)	55.5 (±8.01)	55.593 (±8.01)
Male sex-no. (%)	108,467 (48.59%)	108,509 (48.61%)	108,579 (48.64%)	108,529 (48.61%)	108,458 (48.58%)
Lipid treatment-no./total no. (%)	24,259 (10.87%)	24,246 (10.86%)	19,778 (8.86%)	19,756 (8.85%)	19,774 (8.86%)
Diabetes mellitus-no./total no. (%)	5840 (2.62%)	5829 (2.61%)	5847 (2.62%)	5830 (2.62%)	5837 (2.61%)
Body mass index	26.9 (±4.33)	26.9 (±4.33)	26.9 (±4.34)	26.9 (±4.33)	26.9 (±4.34)
MET Score	2642.4 (±2664.35)	2642.8 (±2665.44)	2642.5 (±2665.28)	2642.1 (±2664.77)	2641.4 (±2663.52)
current smoking-no. (%)	23,593 (10.57%)	23,599 (10.57%)	23,659 (10.60%)	23,553 (10.55%)	23,620 (10.58%)
past smoking-no. (%)	77,860 (34.88%)	77,884 (34.89%)	77,875 (34.88%)	77,881 (34.89%)	77,875 (34.88%)
never smoking-no. (%)	121,130 (54.26%)	121,097 (54.24%)	121,044 (54.22%)	121,148 (54.27%)	121,084 (54.24%)
Systolic blood pressure-mean (SD)-mmHg	140.4 (±19.47)	140.4 (±19.48)	140.4 (±19.48)	140.4 (±19.48)	140.4 (±19.49)
Diastolic blood pressure-mean (SD)-mmHg	83.4 (±10.82)	83.4 (±10.81)	83.4 (±10.81)	83.4 (±10.81)	83.4 (±10.82)
Red wine intake-mean (SD)-glass/week	3.9 (±5.68)	3.9 (±5.68)	3.9 (±5.68)	3.93 (±5.68)	3.9 (±5.68)
White wine intake-mean (SD)-glass/week	2.7 (±4.88)	2.7 (±4.88)	2.7 (±4.88)	2.7 (±4.88)	2.7 (±4.88)
Fortified wine intake- mean (SD)-glass/week	0.2 (±1.21)	0.2 (±1.21)	0.2 (±1.22)	0.2 (±1.22)	0.2 (±1.22)
Beer intake-mean (SD)-pints/week	2.9 (±5.59)	2.9 (±5.59)	2.9(±5.62)	2.9 (±5.60)	2.9 (±5.60)
Spirits intake-mean (SD)-measures/week	1.8 (±5.29)	1.8 (±5.29)	1.8 (±5.32)	1.8 (±5.36)	1.8 (±5.30)

**Table 2:** Overview of the results of Mendelian Randomisation analysis.

	Single Instrument MR			Multiple Instrument MR		
	Beta	95% CI	Observed *p*-Value	Beta	95% CI	Observed *p*-Value
Gamma Glutamyl Transferase	9.7	5.8, 13.6	0.0001	9.4	5.9, 12.9	0
Mean Sphered Cell Volume	0.2	0.15, 0.31	0	0.19	0.11, 027	0.0001
Mean Corpuscular Haemoglobin	0.3	0.18, 0.36	0	0.3	0.19, 0.35	0
Mean Corpuscular Volume	0.3	0.18, 0.36	0	0.3	0.19, 0.36	0
Unplanned physical activity by method of transport	−0.04	−0.07, −0.01	0.01	−0.06	−0.1, −0.03	0.0008
Wholemeal/ wholegrain bread consumption	−0.05	−0.09, −0.02	0.006	−0.06	−0.1, −0.02	0.008
White bread consumption	0.05	0.02, 0.09	0.002	0.05	0.02, 0.09	0.008

**Table 3:** Overview of the rs1229984 PheWAS results in the UK biobank cohort.

Description	Effect Estimate	95% CI	Odds Ratio	*p*-Value
Alcohol-related disorders	0.24	(0.16, 0.32)	1.3	4.87 × 10^−10^
Alcoholism	0.26	(0.16, 0.36)	1.3	2.52 × 10^−8^
Alcoholic liver damage	0.27	(0.15, 0.39)	1.3	3.47 × 10^−6^
Enthesopathy	−0.06	(−0.08, −0.04)	0.9	1.05 × 10^−5^

## Data Availability

Not applicable.

## Supplementary Materials

The following supporting information can be downloaded at: https://www.mdpi.com/article/10.3390/nu14142943/s1, Table S1: Genetic variants used in Mendelian randomisation analysis.
