# Supplementary material for: Molecular Alterations Caused by Alcohol Consumption in the UK Biobank: A Mendelian Randomisation Study
Source: Nutrients. 2022 Jul 19;14(14):2943. doi: 10.3390/nu14142943 (PMC9317105; doi:10.3390/nu14142943)
Supplement: Supplementary file 1 [file nutrients-14-02943-s001.zip › nutrients-1751337-supplementary.pdf]

Molecular Alterations Caused by Alcohol Consumption in the UK Biobank: A Mendelian Randomisation Study

Felix O'Farrell [1], Xiyun Jiang [1], Shahad Aljifri [1] and Raha Pazoki [1,2,*]

1 Division of Biomedical Sciences, Department of Life Sciences, College of Health and Life Sciences, Brunel University London, Uxbridge UB8 3PH, UK

2 Department of Epidemiology and Biostatistics, School of Public Health, St Mary's Campus, Norfolk Place, London W2 1PG, UK

* Correspondence: author: raha.pazoki@brunel.ac.uk

Table S1. Genetic variants used in Mendelian randomisation analysis.

SNP	effect allele	other allele	effect allele frequency	p value	effect estimate	standard error	sample size	F-statistics	SNP strength
rs705687	A	G	0.796	2.2×10^{-01}	-0.004	0.004	221645	1.501	Weak
rs58107686	C	A	0.344	3.1×10^{-04}	-0.013	0.004	186537	12.960	Strong
rs12088813	A	C	0.26	2.3×10^{-01}	-0.004	0.003	223144	1.472	Weak
rs5024204	A	T	0.267	2.0×10^{-02}	0.008	0.003	220093	5.402	Weak
rs10753661	G	A	0.702	2.7×10^{-02}	-0.007	0.003	219744	4.909	Weak
rs28680958	G	A	0.23	3.2×10^{-04}	-0.013	0.004	221508	12.991	Strong
rs823114	G	A	0.553	1.1×10^{-03}	0.010	0.003	220432	10.633	Strong
rs77165542	C	T	0.0283	1.3×10^{-01}	-0.013	0.009	190275	2.287	Weak
rs1260326	T	C	0.595	4.7×10^{-06}	0.014	0.003	221214	20.796	Strong
rs2178197	A	G	0.542	4.6×10^{-01}	-0.002	0.003	224519	0.553	Weak
rs13383034	C	T	0.336	2.0×10^{-04}	0.012	0.003	219150	13.762	Strong

SNP	effect allele	other allele	effect allele frequency	p value	effect estimate	standard error	sample size	F-statistics	SNP strength
rs13032049	A	G	0.28	1.4×10^{-06}	0.016	0.003	219119	23.225	Strong
rs828867	G	A	0.544	2.3×10^{-02}	0.007	0.003	215058	5.157	Weak
rs11692435	G	A	0.0856	1.8×10^{-01}	0.007	0.005	197923	1.789	Weak
rs13024996	C	A	0.352	4.8×10^{-03}	-0.009	0.003	221113	7.967	Weak
rs72859280	G	T	0.0355	2.7×10^{-05}	0.035	0.008	210722	17.638	Strong
rs56337305	T	C	0.375	5.0×10^{-04}	-0.011	0.003	219788	12.000	Strong
rs13094887	A	T	0.286	3.7×10^{-02}	-0.007	0.003	220966	4.335	Weak
rs62250685	A	G	0.612	5.5×10^{-05}	-0.012	0.003	220967	16.208	Strong
rs13066454	C	T	0.401	1.4×10^{-01}	-0.005	0.003	219462	2.216	Weak
rs9838144	G	C	0.194	7.4×10^{-02}	-0.007	0.004	217833	3.208	Weak
rs2011092	T	C	0.314	6.1×10^{-01}	-0.002	0.003	223636	0.253	Weak
rs60654199	C	A	0.067	6.6×10^{-04}	-0.022	0.006	216549	11.639	Strong
rs6787172	T	G	0.546	8.5×10^{-05}	-0.012	0.003	222544	15.312	Strong
rs3748034	G	T	0.133	5.9×10^{-05}	-0.017	0.004	196985	16.038	Strong
rs7682824	C	T	0.217	4.2×10^{-03}	-0.010	0.003	123149	8.216	Weak
rs11940694	A	G	0.595	4.8×10^{-14}	0.023	0.003	222804	56.745	Strong
rs4501255	C	G	0.236	6.7×10^{-03}	0.009	0.004	221053	7.352	Weak
rs12499107	A	G	0.123	5.2×10^{-01}	0.003	0.004	219969	0.410	Weak
rs1229984	T	C	0.953	1.1×10^{-65}	0.145	0.008	203476	294.457	Strong
rs10028756	G	A	0.126	1.5×10^{-01}	-0.007	0.005	216539	2.125	Weak
rs36052336	A	G	0.0519	1.0×10^{-02}	-0.016	0.006	215241	6.652	Weak
rs2165670	G	A	0.0984	2.0×10^{-08}	0.026	0.005	221948	31.517	Strong
rs79139602	A	T	0.0339	4.6×10^{-01}	0.008	0.010	220441	0.544	Weak

SNP	effect allele	other allele	effect allele frequency	p value	effect estimate	standard error	sample size	F-statistics	SNP strength
rs4699791	G	A	0.114	6.4×10^{-01}	0.002	0.005	216148	0.215	Weak
rs13107325	C	T	0.0654	1.3×10^{-07}	-0.035	0.007	217038	27.784	Strong
rs4690727	C	G	0.728	3.9×10^{-04}	0.012	0.003	224095	12.724	Strong
rs10004020	G	A	0.705	3.8×10^{-01}	0.003	0.003	224239	0.773	Weak
rs12651313	C	G	0.463	4.7×10^{-03}	-0.008	0.003	223005	7.987	Weak
rs4916723	A	C	0.404	2.0×10^{-03}	-0.009	0.003	219877	9.587	Weak
rs12655091	G	A	0.508	2.8×10^{-04}	-0.011	0.003	223535	13.135	Strong
rs55872084	G	T	0.218	2.2×10^{-08}	0.020	0.004	222708	31.247	Strong
rs10085696	A	G	0.201	1.6×10^{-04}	-0.015	0.004	225774	14.258	Strong
rs6460047	T	C	0.203	6.4×10^{-02}	0.007	0.004	215693	3.432	Weak
rs10236149	A	G	0.17	3.0×10^{-03}	-0.014	0.005	220984	8.779	Weak
rs35034355	G	A	0.486	2.1×10^{-03}	-0.009	0.003	224533	9.469	Weak
rs6951574	T	C	0.459	3.5×10^{-03}	0.009	0.003	207047	8.505	Weak
rs13250583	C	T	0.204	8.0×10^{-03}	-0.010	0.004	224238	7.038	Weak
rs1217091	T	C	0.81	3.9×10^{-03}	0.011	0.004	218773	8.356	Weak
rs28601761	C	G	0.405	4.4×10^{-05}	0.012	0.003	218148	16.588	Strong
rs55932213	A	G	0.701	9.3×10^{-03}	0.009	0.003	218281	6.757	Weak
rs10978550	T	C	0.205	7.8×10^{-02}	-0.006	0.004	223278	3.101	Weak
rs7074871	G	A	0.254	3.2×10^{-03}	-0.010	0.003	225774	8.721	Weak
rs17665139	C	T	0.144	3.5×10^{-03}	-0.012	0.004	225644	8.519	Weak
rs7950166	C	T	0.614	2.4×10^{-03}	-0.009	0.003	224871	9.235	Weak
rs11030084	C	T	0.198	3.8×10^{-03}	-0.011	0.004	224959	8.395	Weak
rs56030824	G	A	0.317	5.0×10^{-05}	-0.013	0.003	219170	16.449	Strong

SNP	effect allele	other allele	effect allele frequency	p value	effect estimate	standard error	sample size	F-statistics	SNP strength
rs10750025	C	T	0.698	2.7×10^{-04}	0.012	0.003	220854	13.325	Strong
rs1713676	A	G	0.522	1.9×10^{-02}	-0.007	0.003	223561	5.502	Weak
rs4938230	C	A	0.837	3.9×10^{-03}	0.012	0.004	222828	8.361	Weak
rs682011	T	C	0.567	8.7×10^{-03}	0.008	0.003	222378	6.899	Weak
rs12795042	A	C	0.621	2.3×10^{-05}	-0.013	0.003	214048	17.973	Strong
rs10876188	C	T	0.466	7.1×10^{-03}	-0.008	0.003	224376	7.231	Weak
rs3809162	A	G	0.414	2.3×10^{-03}	0.009	0.003	218440	9.299	Weak
rs10506274	G	T	0.458	1.1×10^{-02}	-0.008	0.003	224459	6.487	Weak
rs4842786	G	A	0.588	1.8×10^{-06}	-0.015	0.003	220405	22.750	Strong
rs500321	A	T	0.729	1.2×10^{-03}	-0.011	0.003	220776	10.654	Strong
rs1123285	C	G	0.339	1.7×10^{-03}	-0.010	0.003	221889	9.920	Weak
rs2180870	T	C	0.132	1.5×10^{-04}	-0.017	0.004	218628	14.358	Strong
rs28929474	C	T	0.0154	1.9×10^{-03}	-0.036	0.012	217304	9.626	Weak
rs11625650	G	A	0.24	4.9×10^{-03}	-0.010	0.004	180176	7.959	Weak
rs2472297	C	T	0.206	9.4×10^{-03}	0.009	0.003	204466	6.769	Weak
rs12907323	A	G	0.423	8.9×10^{-03}	0.008	0.003	220126	6.850	Weak
rs2764771	G	A	0.324	7.8×10^{-03}	0.008	0.003	221589	7.067	Weak
rs17177078	C	T	0.0664	2.0×10^{-03}	-0.019	0.006	219671	9.585	Weak
rs378421	G	A	0.405	3.7×10^{-07}	-0.015	0.003	211061	25.832	Strong
rs113443718	G	A	0.282	9.7×10^{-03}	-0.008	0.003	201951	6.667	Weak
rs62044525	C	G	0.172	3.2×10^{-05}	-0.016	0.004	219063	17.202	Strong
rs7185555	G	C	0.141	5.6×10^{-05}	-0.017	0.004	218108	16.228	Strong
rs79616692	G	C	0.11	1.1×10^{-03}	0.016	0.005	220007	10.619	Strong

SNP	effect allele	other allele	effect allele frequency	p value	effect estimate	standard error	sample size	F-statistics	SNP strength
rs1104608	G	C	0.418	1.8×10^{-02}	-0.007	0.003	207406	5.590	Weak
rs4548913	G	A	0.621	1.7×10^{-01}	-0.004	0.003	216859	1.911	Weak
rs3803800	A	G	0.772	4.6×10^{-03}	0.010	0.004	215507	7.959	Weak
rs2854334	A	G	0.608	8.8×10^{-05}	0.012	0.003	220715	15.323	Strong
rs10438820	C	T	0.685	3.0×10^{-04}	0.012	0.003	224723	13.102	Strong
rs9950000	C	T	0.411	2.6×10^{-02}	-0.007	0.003	224879	5.000	Weak
rs4092465	A	G	0.61	4.9×10^{-05}	-0.013	0.003	210391	16.578	Strong
rs281379	G	A	0.477	6.1×10^{-09}	0.017	0.003	218248	33.930	Strong
rs4815364	G	A	0.628	1.8×10^{-02}	0.007	0.003	214401	5.556	Weak
rs9607814	C	A	0.212	1.1×10^{-01}	-0.007	0.004	186672	2.580	Weak
